# Ultra-Sensitive Flexible Pressure Sensor Based on Microstructured Electrode

**DOI:** 10.3390/s20020371

**Published:** 2020-01-09

**Authors:** Mengmeng Li, Jiaming Liang, Xudong Wang, Min Zhang

**Affiliations:** 1 Shenzhen International Graduate School, Tsinghua University, Shenzhen 518055, China; li-mm17@mails.tsinghua.edu.cn (M.L.); wangxd15@mails.tsinghua.edu.cn (X.W.); 2 Tsinghua-Berkeley Shenzhen Institute, Tsinghua University, Shenzhen 518055, China; liangjm16@mails.tsinghua.edu.cn

**Keywords:** pressure sensor, flexible, capacitive, pyramidal microstructure

## Abstract

Flexible pressure sensors with a high sensitivity in the lower zone of a subtle-pressure regime has shown great potential in the fields of electronic skin, human–computer interaction, wearable devices, intelligent prosthesis, and medical health. Adding microstructures on the dielectric layer on a capacitive pressure sensor has become a common and effective approach to enhance the performance of flexible pressure sensors. Here, we propose a method to further dramatically increase the sensitivity by adding elastic pyramidal microstructures on one side of the electrode and using a thin layer of a dielectric in a capacitive sensor. The sensitivity of the proposed device has been improved from 3.1 to 70.6 kPa^−1^ compared to capacitive sensors having pyramidal microstructures in the same dimension on the dielectric layer. Moreover, a detection limit of 1 Pa was achieved. The finite element analysis performed based on electromechanical sequential coupling simulation for hyperelastic materials indicates that the microstructures on electrode are critical to achieve high sensitivity. The influence of the duty ratio of the micro-pyramids on the sensitivity of the sensor is analyzed by both simulation and experiment. The durability and robustness of the device was also demonstrated by pressure testing for 2000 cycles.

## 1. Introduction

Flexible pressure sensors have drawn a tremendous amount of attention due to their wide applications in mobile biomonitoring, wearable electronics, artificial intelligence, energy harvesting and human interface devices [1,2,3,4,5]. The pressure involved with most of these applications is mainly divided into four ranges, i.e., ultra-low pressure (<1 Pa), subtle-pressure regime (1 Pa–1 kPa), low-pressure regime (1 kPa–10 kPa), and medium pressure regime (10 kPa–100 kPa) [4,6]. Flexible pressure sensors in the subtle-pressure regime with high sensitivity are needed for the in situ detection of weak physiological signals such as blood pressure, pulse wave, heart sound, and breath sound. The key parameters that evaluate flexible pressure sensors include sensitivity, response time, detection limit, and reliability. Various successful pressure-sensing mechanisms have been demonstrated, including capacitive [6,7,8], piezoresistive [5,9,10,11], piezoelectric [12,13], optical [14], and triboelectric principles [15]. Capacitive pressure sensors, taking advantage of their simple device construction, long-term drift stability, and low power consumption, have been a better choice for electronic skin and wearable devices [16,17,18].

In recent years, various approaches have been investigated to improve the performance of capacitive flexible pressure sensors. A typical capacitive pressure sensor is composed of a dielectric layer sandwiched by two electrode plates. The capacitance of it is mainly determined by the facing area and distance of the two electrode plates, and the permittivity of the dielectric layer in between. The electrode plates and the dielectric layer need to be flexible for a flexible pressure sensor. In order to improve the sensitivity of the flexible sensor to pressure, the dielectric layer should be as soft as possible. Therefore, microstructures in the dielectric layer have been extensively used to lower the Young’s modulus. For example, Kwon and co-workers used a microporous dielectric elastomer molded from a sugar cube as the dielectric layer of a capacitive sensor, and realized highly sensitive and stable pressure sensing with the sensitivity of 0.6 kPa^−1^ [18]. Bao et al. fabricated polydimethylsiloxane (PDMS) pyramidal microstructures using wet etched silicon mold to develop a highly sensitive capacitive pressure sensor with a sensitivity of 0.55 kPa^−1^ [6]. Kim et al. fabricated a capacitive pressure sensor with nano-needle structures on the dielectric layer [19]. Zhang et al. used microstructured PDMS molded from natural leaves as the dielectric layer for a flexible pressure sensor and demonstrated a high sensitivity of 19.8 kPa^−1^ in the pressure range below 0.3 kPa [20].

Although a microstructured dielectric layer in a capacitive sensor can enhance the sensitivity to pressure, it also increases the thickness of a dielectric layer and hence the distance of the electrode plates. The capacitance of a parallel-plate capacitor can be expressed as:

(1) C∝Aε/d,

where *C* is the capacitance, *ε* is the permittivity of dielectric layers, *A* is the effective area of the plates, and *d* is the distance between the plate electrodes. As the capacitance *C* is inversely proportional to the plate distance *d*, *C* changes much faster in the range where *d* is small than in the range where *d* is large. Therefore, a conventional capacitive sensor is always designed to work in the small *d* region so that a high sensitivity can be achieved. However, dielectric elastomers in thin flexible capacitive sensors are not normally used for highly sensitive applications due to the relatively high Young’s modulus of the commonly used elastomers such as PDMS, polyurethane, and Ecoflex. Moreover, several micrometer thick elastomer films exhibit significant viscoelasticity, which deteriorates the response speed of the device [21].

A possible way to further improve the sensitivity is to fabricate microstructures on the electrode instead of on the dielectric layer. In this case, a thin dielectric layer can be used so that the initial electrode distance can be much smaller compared to sensors with a microstructured dielectric layer (SMD). Cheng and co-workers fabricated a capacitive sensor with a PDMS microstructured electrode to obtain both high sensitivity and low hysteresis [22]. A sensitivity of 3.73 KPa^−1^ and hysteresis of 4.42% were observed. Since the thickness of the dielectric layer (6 μm) is still large compared to the height of the pyramid structures (~21 μm), the relative distance change of the two electrodes and, consequently, the capacitance change under pressure are not very high and can be further improved. In this work, we demonstrate a highly sensitive flexible capacitive pressure sensor with a micro-pyramidal elastomer electrode plate. Gold film was deposited on the PDMS micro-pyramidal structures to act as a microstructured electrode. By using a 1-μm parylene as the dielectric layer, an ultra-high sensitivity of 70.6 kPa^−1^ was achieved compared with SMD of the same dimensions.

## 2. Materials and Methods

### 2.1. Fabrication of Sensors with Microstructured Electrode (SME) and SMD

**Figure 1:**
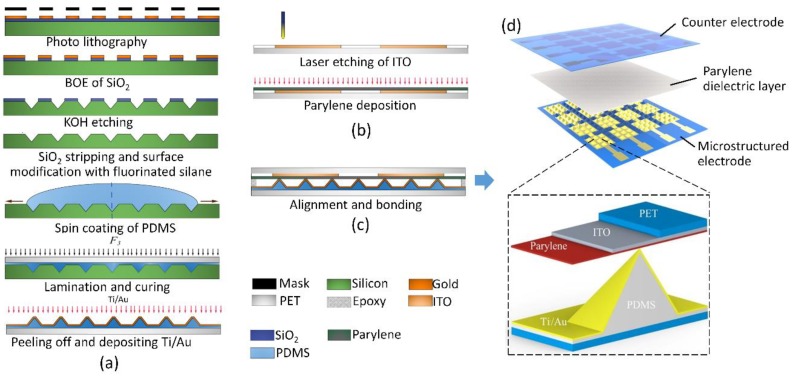
Fabrication process of the (**a**) microstructured electrode, (**b**) counter electrode, and (**c**) final sensor array; (**d**) illustration of the capacitive pressure sensor based on a microstructured electrode.

The fabrication process of the proposed flexible pressure sensor is illustrated in Figure 1. The PDMS pyramid arrays with different duty ratios, that is, the edge length of pyramid divided by spacing between pyramids, are fabricated using wet-etched silicon molds and a lamination method. A (100) oriented silicon wafer with a 300 nm thermally grown oxide was patterned using standard photolithography with positive photoresist S1813. The oxide was etched by using a 6:1 buffered oxide etch (BOE) solution followed by ultrasonic cleaning in the deionized (DI) water for 5 min. The BOE solution is composed of hydrofluoric acid (4 mol/L) and ammonium fluoride (15 mol/L. The substrate silicon was then etched by 30% potassium hydroxide (KOH) at 80 °C. After a pyramid pits array was fully generated by KOH etching, the wafer was sonicated in the DI water for 15 min, and BOE etched again to remove the SiO_2_ residual. The fabricated silicon cavities were then treated with vapor phase 1*H*,1*H*,2*H*,2*H*-perfluorodecyltrichlorosilane to lower the surface energy and hence the adhesion force between PDMS and silicon mold.

A 50 μm polyethylene terephthalate (PET) film was first rinsed with ethanol for 5 min and sonicated in deionized water for 10 min. Then, the film was blown dry with a nitrogen flow and further dried in an oven for 30 min at 120 °C. A 10:1 mixture of PDMS elastomer (Sylgard 184, Dow Corning, Midland, TX, USA) to a crosslinking agent was spin coated on the fabricated silicon mold at 8000 rpm, and the cleaned PET film activated by oxygen plasma at 90 W for 90 s was laminated on top of the uncured PDMS film. The surface activation of PET improves the adhesion between PET and PDMS microstructure layers.

The lamination was clamped at a pressure over 12 MPa for 15 min at room temperature and then heated to 80° for at least 3 h under the same pressure. The cured PDMS pyramid microstructures were then peeled off together with a PET substrate from the silicon mold, and a metal conductive layer (Ti/Au, 5 nm/60 nm) was evaporated through a shadow mask on the surface of PDMS microstructures. In order to enhance the adhesion force between metal layers and PDMS substrate, a heavy oxygen plasma etching was conducted at 90 W for 300 s before metal evaporation.

An indium tin oxide (ITO) electrode on a PET substrate patterned by laser etching was used as the counter electrode of the device. A 1 μm parylene C thin film was deposited on the ITO electrodes to serve as the dielectric layer. The capacitive sensor is finally fabricated by bonding the microstructured electrode layer with the counter electrode by resin epoxy on the edges.

Capacitive pressure sensors with flat electrodes and microstructured dielectric layer were also fabricated for comparison. The PDMS layer with pyramid array was fabricated using the same silicon mold mentioned above. Two ITO electrodes on a PET substrate were then bonded on the two sides of the PDMS layer to form a capacitive sensor.

### 2.2. Characterization and Measurements

**Figure 2:**
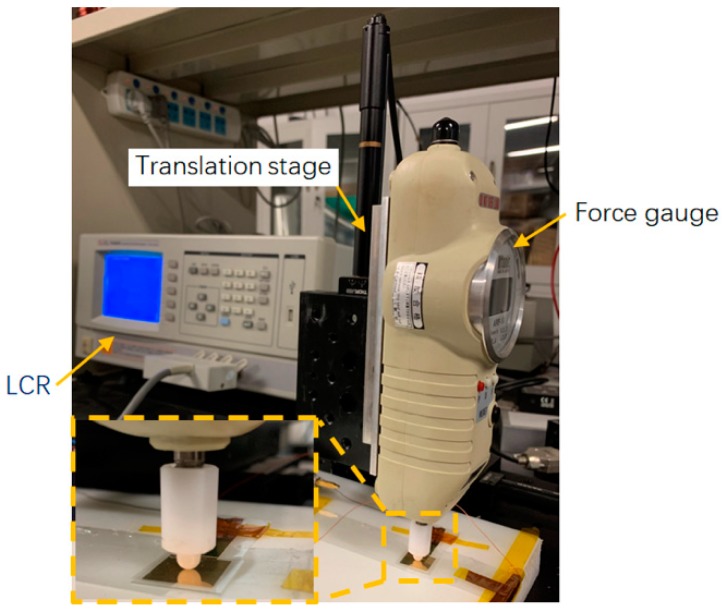
Photograph of the full experimental setup.

The microstructures of the samples were inspected by a field-emission scanning electron microscopy (SU8010, Hitachi High-Technologies Corporation. Tokyo, Japan) operated at 5 kV. The external pressure was measured by a force gauge (M5-05 MARK-10, Quality Control Solutions, Inc. Temecula, CA, USA) with 0.5 mN resolution. A computer-controlled three-axis translation stage (PT101(M), Thorlabs, Inc. Newton, MA, USA) with 50-nm resolution was used to apply the pressure precisely. The translation stage is driven by direct current (DC) servo motors and the displacement is controlled by a built-in displacement sensor. The displacement of the compression on the pyramids was recorded through the readings of the translation stage. The capacitance was measured using an inductance capacitance and resistance (LCR) meter (TH2828S, Tonghui Electronics, Changzhou, China) with a testing frequency of 1 kHz. The test setup of the sensors is shown in Figure 2.

A rice seed with 24 mg was used to investigate the detection limit of SME. A small piece of glass slide (170 μm thick) was placed on the sensor before loading the rice seed. The size of the glass slide is the same as a unit of the sensor, which can ensure that the weight of the rice seed transfers to the surface of the sensor unit uniformly. The pressure can be calculated by dividing the weight of rice seed with the area of the sensor unit.

### 2.3. Finite Element Anylasis (FEA) of the Compression Process of PDMS Pyramids

The mechanical behavior of PDMS is assumed to be nonlinear in the full strain range from −55% (compression) to 50% (tensile) [23]. The constitutive equation of hyperelastic incompressible materials usually can be described by means of the Mooney–Rivlin (M–R) strain energy density function W. To be simplified and practical convenient use, it can be expressed as a two-parameter M-R model:

(2) $$W=C_{10}\left({\bar{I}}_{1}-2\right)+C_{01}\left({\bar{I}}_{2}-3\right),$$

where *C*_10_ and *C*_01_ are M-R material constants, ${\bar{I}}_{1}$ and ${\bar{I}}_{2}$ the first and second strain invariants of the unimodular component of the left Cauchy–Green deformation tensor, respectively.

For the uniaxial compression, the strain invariants can be expressed by:

(3) $${\bar{I}}_{1}=\lambda^{2}+\frac{2}{\lambda },$$

(4) $${\bar{I}}_{2}=\frac{1}{\lambda^{2}}+2\lambda ,$$

where *λ* = 1 + *ε*, and *ε* is the normal strain.

As a hyperplastic material, PDMS is assumed to be incompressible or nearly incompressible and its Poisson’s ratio is around 0.495. When *λ*→1, the initial Young’s modulus can be described as follows:

(5) $$E=\underset{\lambda \to 1}{\lim}\frac{\partial W}{\partial^{2}\lambda }=6\left(C_{10}+C_{01}\right),$$

where *E* is the Young’s modulus of the polymer.

The Young’s modulus of PDMS is affected by the curing temperature and time and the ratio of the polymer and crosslinking agent. The Young’s modulus of the PDMS used in this work was measured experimentally by an electro-mechanical universal testing machine (CMT4204, American Industrial Systems Co., Ltd. Irvine, CA, USA). The elastic Young’s modulus of 10:1 PDMS is 3.3 MPa in the compression condition and 2.16 MPa in the extension condition (Figure S1). Based on the measured Young’s modulus of PDMS, the coefficients of the M–R constitutive equation, i.e., *C*_10_ and *C*_01_ can be calculated using Mathematica programming.

A 3D finite element modeling was developed to simulate the elastically deform process of pyramid under external pressure. The simulations of the deformation were run in the commercial finite element code ANSYS Workbench and the calculations of the capacitance were conducted using MATLAB. Given the central symmetry of the problem, only 1/8 of the 3D symmetrical finite element model of a pyramid unit is constructed, and their symmetrical face was applied frictionless support. The upper plate was applied boundary displacement conditions at the interlayer of the sensor, resulting in a 60% change compared with their original distance, while the lower plate was imposed on the constraints of fixed support (Figure S2).

**Figure 3:**
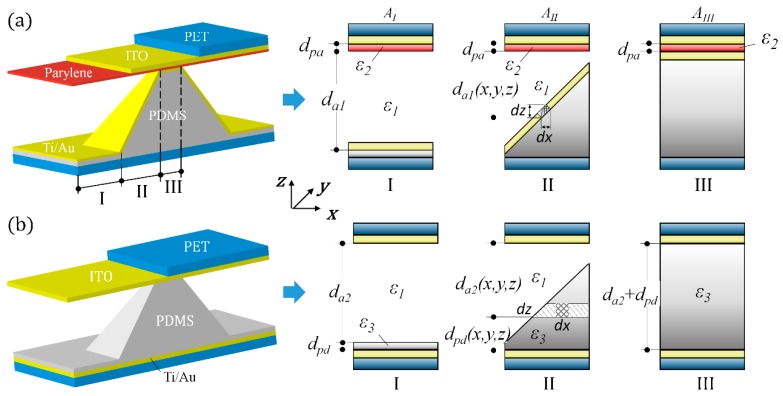
Illustration and capacitance calculation principle of a single unit of (**a**) SME and (**b**) SMD, where *A_I_*, *A_II_*, and *A_III_* are the electrode area of regions I, II, and III, respectively, *ε*_1_, *ε*_2_, and *ε*_3_ the permittivity of air, parylene, and PDMS, respectively, *d_a_*_1_ the thickness of air dielectric at the blank region I in SME, *d_a_*_2_ the thickness of air dielectric at the blank region I in SMD, *d_pa_* the thickness of parylene layer, *d_pd_* the thickness of PDMS layer at the blank region I in SMD, *d_a_*_1_(*x,y,z*) the thickness of air dielectric at the slope region II in SME, *d_a_*_2_(*x,y,z*) the thickness of air dielectric at the slope region II in SMD, *d_pd_*(*x,y,z*) the thickness of PDMS at the slope region II in SMD.

After FEA simulation, the node unit information was first derived from ANSYS. The surface nodes information of the deformed pyramid were then extracted using MATLAB programming to calculate the capacitance for SME and SMD. The calculation principle of the capacitance for SME and SMD is shown in Figure 3. There are three regions in a compressed pyramid unit for capacitance calculation, that is, the blank region I, the slope region II, and the contact region III. For the flat contact region and blank region, the parallel plate model can be used directly. For the slope region, integration was used to adopt the parallel plate model on a curved surface. The area of *dx* × *dy* is small enough that the area can be treated as a flat surface approximately and, therefore, the parallel plate model still works. The total capacitance of the pyramid unit can be calculated by summing the capacitance of the three regions. For SME, the capacitance of a pyramid unit is

(6) $$C_{SME}=C_{I}+C_{II}+C_{III}$$

and

(7) $$C_{I}=\frac{\frac{A_{I}\varepsilon_{1}}{d_{a1}}\times \frac{A_{I}\varepsilon_{2}}{d_{pa}}}{\frac{A_{I}\varepsilon_{1}}{d_{a1}}+\frac{A_{I}\varepsilon_{2}}{d_{pa}}},$$

(8) $$C_{II}=\frac{∭\frac{A_{II}\varepsilon_{1}}{d_{a1}\left(x,y,z\right)}dxdydz\times \frac{A_{II}\varepsilon_{2}}{d_{pa}}}{∭\frac{A_{II}\varepsilon_{1}}{d_{a1}\left(x,y,z\right)}dxdydz+\frac{A_{II}\varepsilon_{2}}{d_{pa}}},$$

(9) $$C_{III}=\frac{A_{III}\varepsilon_{2}}{d_{pa}},$$

where *A_I_*, *A_II_*, and *A_III_* are the electrode area of regions I, II, and III, respectively, *ε*_1_, *ε*_2_ the permittivity of air and parylene, respectively, *d_a_*_1_ the thickness of air dielectric at the blank region I in SME, *d_pa_* the thickness of parylene layer, and *d_a_*_1_(*x,y,z*) the thickness of air dielectric at the slope region II in SME.

For SMD, the capacitance of a pyramid unit is

(10) $$C_{SMD}=C_{I}^{\prime }+C_{II}^{\prime }+C_{III}^{\prime }$$

and

(11) $$C_{I}^{\prime }=\frac{\frac{A_{I}\varepsilon_{1}}{d_{a2}}\times \frac{A_{I}\varepsilon_{3}}{d_{pd}}}{\frac{A_{I}\varepsilon_{1}}{d_{a2}}+\frac{A_{I}\varepsilon_{3}}{d_{pd}}},$$

(12) $$C_{II}^{\prime }=\frac{∭\frac{A_{II}\varepsilon_{3}}{d_{pd}\left(x,y,z\right)}dxdydz\times ∭\frac{A_{II}\varepsilon_{1}}{d_{a2}\left(x,y,z\right)}dxdydz}{∭\frac{A_{II}\varepsilon_{3}}{d_{pd}\left(x,y,z\right)}dxdydz+∭\frac{A_{II}\varepsilon_{1}}{d_{a2}\left(x,y,z\right)}dxdydz},$$

(13) $$C_{III}^{\prime }=\frac{A_{III}\varepsilon_{3}}{d_{a2}+d_{pa}},$$

where *ε*_3_ is the permittivity of PDMS, *d_a_*_2_ the thickness of air dielectric at the blank region I in SMD, *d_pd_* the thickness of PDMS layer at the blank region I in SMD, *d_a_*_2_(*x,y,z*) the thickness of air dielectric at the slope region II in SMD, and *d_pd_(x,y,z)* the thickness of PDMS at the slope region II in SMD.

## 3. Results and Discussion

The overall structure of SME is shown in Figure 3a. The sensor is composed of an ITO counter electrode, a 1 μm parylene dielectric layer, and a PDMS microstructured electrode coated with a thin layer of Ti/Au (5 nm/60 nm). The structure of SMD is also shown in Figure 3b for comparison. The illustration on the right side explains that the initial capacitance of SME is smaller than SMD due to the smaller initial electrode distance as well as a smaller permittivity. Since the dimensions of the PDMS pyramids are the same for two types of devices, the deformation of the pyramids is the same under the same pressure. The smaller initial capacitance of SME leads to a larger relative capacitance change and thus a higher sensitivity.

**Figure 4:**
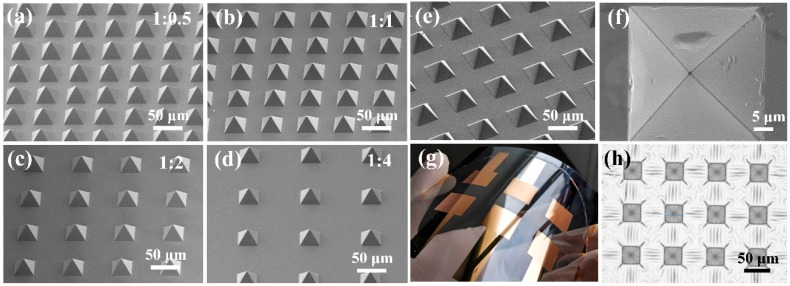
SEM images of a PDMS pyramid array with duty ratio of (**a**) 1:0.5, (**b**) 1:1, (**c**) 1:2, and (**d**) 1:4, (**e**) SEM image of the PDMS pyramid coated with Ti/Au, (**f**) a detailed image of the Ti/Au coated PDMS pyramid, indicating that the metal film deposited on PDMS structures uniformly without cracks or wrinkles, (**g**) the fabricated device bent by hand, (**h**) photo image of the wrinkles on the PDMS pyramid array coated with Ti/Au when there is no PET backup during metal deposition.

The fabricated PDMS pyramid arrays with different duty ratios from 1:4 to 1:0.5 are shown in Figure 4. The edge length of the pyramid microstructures is 35 μm and the height is 24.7 μm for all of the samples. As shown in Figure 4e,f, a smooth and uniform Ti/Au thin film was coated on the PDMS pyramid structures. Metal thin films deposited on PDMS substrate usually exhibit wrinkles and cracks due to the different thermal expansion rate between metals and PDMS [24,25]. Our experiment also shows that wrinkles occur on the PDMS microstructures (Figure 4h). This problem was solved in our work by bonding a PET substrate with an ITO electrode on the PDMS film during the metal evaporation process. The thermal expansion of the PDMS structure was eliminated by the PET substrate with a lower thermal expansion rate and higher Young’s modulus.

**Figure 5:**
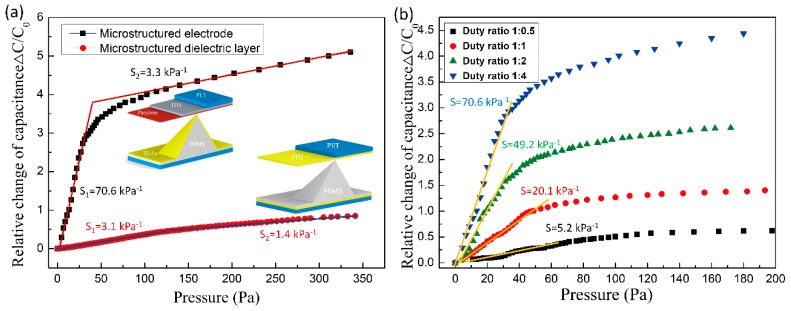
Performance of the flexible pressure sensor. (**a**) pressure–response curves for a CME and CMD with the same pyramid dimensions, (**b**) effect of pyramid duty ratio on the sensitivity of CME.

The sensing performance of fabricated sensors is evaluated in Figure 5. As shown in Figure 5a, two linear regions of the relative capacitance change (Δ*C*/*C*_0_) as a function of applied pressure were observed for both SME and SMD. The relative capacitance change of SME increased rapidly in the pressure regime less than 50 Pa and the sensitivity of the device reaches 70.6 kPa^−1^. The corresponding compression displacement on the pyramids is 9.8 μm. When the applied pressure is higher than 50 Pa, the output of the device increased slower and reached a second linear region. The sensitivity of SME in this region is 3.3 kPa^−1^ and the corresponding displacement is 13.2 μm. By contrast, the device of SMD shows much lower sensitivity of 3.1 and 1.4 kPa^−1^ in the pressure region below and above 50 Pa, respectively. As the dimension of the PDMS pyramids are the same for the two types of sensors, the enhancement of the sensitivity is mainly due to the change of the electrode location from under the PDMS pyramid to on the surface of the PDMS microstructures.

The effect of pyramid density on the performance of the device was also investigated. As shown in Figure 5b, the sensitivity of SME increased dramatically from 5.2 kPa^−1^ to 70.6 kPa^−1^ as the duty ratio of the pyramid structure decreased from 1:0.5 to 1:4. This is because the smaller the duty ratio of pyramid microstructures, the larger the distance change between the upper and lower electrodes when subjected to the same force. Further decrease duty ratio leads to a very large deformation of PDMS pyramids at small pressure and, consequently, rapid saturation of the device.

**Figure 6:**
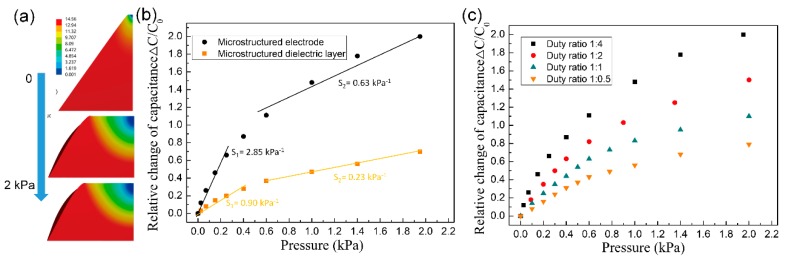
Simulation results of the elastically deform process of the PDMS pyramid (**a**) and sensitivity of a CME and CMD with the same pyramid dimensions (**b**), (**c**) effect of the pyramid density on the sensitivity of CME.

In order to understand the mechanism of the effect of microstructured electrode on sensing performance, FEA simulating the elastically deform process of the PDMS pyramid electrode under external pressure was conducted (Figure 6a). The capacitance change was calculated by extracting the surface nodes information of the deformed pyramid using MATLAB. For comparison, capacitance change of SMD with the same pyramid dimension was also calculated. As shown in Figure 6b, the simulation results show that the relative capacitance change of both SME and SMD have two linear regions as a function of applied pressure and SME has a much higher sensitivity than SMD. In addition, the sensitivity of the device increased with the decrease of pyramid duty ratio (Figure 6c). These results are in agreement with the experiment results qualitatively. However, the sensitivities from the simulation results are much lower than the experimental results, probably because the hypothesis conditions for simulation are not consistent with the real physical situations completely.

**Figure 7:**
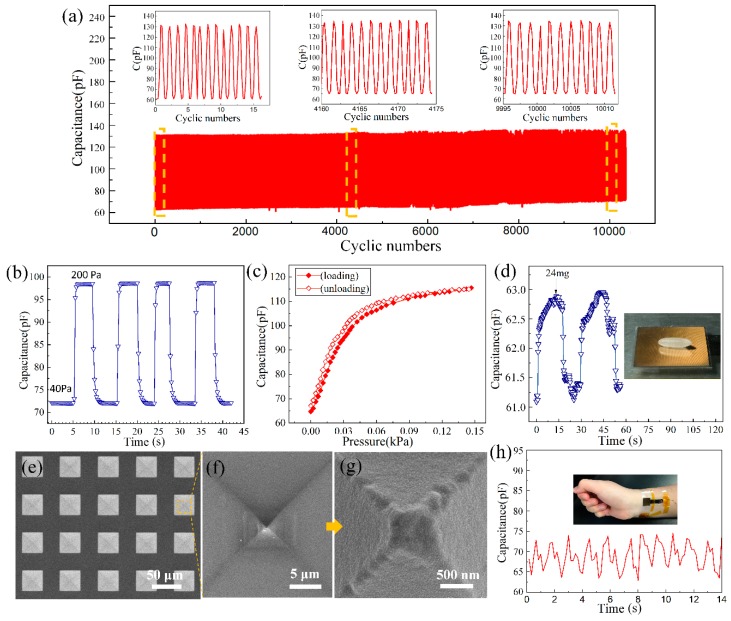
(**a**) Durability test under the pressure of 0–300 Pa, (**b**) stability and (**c**) hysteresis test of the sensor, (**d**) transient response to the application and removal of a 24-mg-weight rice seed on the sensor, the corresponding pressure is 1 Pa, (**e**) SEM image of the pyramid microstructures after pressure tests, indicating the robustness of the metal coating under pressure. (**f**) and (**g**) are the detailed image of the Ti/Au-coated PDMS tip after pressure tests, the square stains are generated under different pressures, (**h**) real-time detection of the wrist pulse signal.

To evaluate the durability of the sensors, we tested the real-time response of the device by repeatedly loading/unloading pressure pulses from 0–300 Pa for 10,200 times. As shown in Figure 7a, the device maintains its function well and no obvious fatigue is observed. The stability of the sensor was tested by applying pressure pulse from 40 to 200 Pa. As shown in Figure 7b, a stable performance was demonstrated. The hysteresis of the sensor was also tested and an 11% hysteresis was observed (Figure 7c). Because of viscoelasticity, the hysteresis of flexible sensor composed of PDMS layers is always high, while the hysteresis of sensors with pyramidal PDMS microstructures is mainly caused by the interfacial adhesion between PDMS structures and the counter layer. Cheng et al. achieved a very low hysteresis of 4.42% by using hierarchically pyramidal PDMS microstructures [22]. The moderate hysteresis of our device is probably because only a small volume of the pyramid tips was used under the pressure range tested. Moreover, as parylene is often used as anti-adhesive layer in the PDMS molding process [26,27], the interfacial adhesion between PDMS and parylene is much lower than that of two PDMS layers. This can also reduce the hysteresis of our device. Increasing the density of the PDMS pyramids will lead to a smaller deformation of PDMS structures at the same pressure compared with low-density structure. Consequently, a smaller adhesion between PDMS and the parylene layer and, therefore, a lower hysteresis is obtained at the cost of a lower sensitivity.

Furthermore, to investigate the detection limit of the microstructured-electrode pressure sensor, a rice seed (24 mg or 1 Pa) was placed on the surface of the sensor, the recorded sensor response is shown in Figure 7d. A capacitance change around 2 pF was observed as the rice seed was loaded and unloaded on the sensor surface, indicating that a 1 Pa detection limit is achieved.

The SEM image of the pyramid structures after pressing is shown in Figure 7e–g. Square imprint stains can be found on the tip region of the pyramid, which were generated under tests with different pressure ranges. A detailed SEM image taken under 40,000 magnification (Figure 7g) reveals that no cracks or exfoliation occur in the metal film. The robustness of the metal film under pressure is probably because of the micro-roughness on the PDMS surface created by the heavy oxygen plasma etching before metal evaporation, which enhances the adhesion force between metal and PDMS.

As a demonstration, the weak human pulse signal could be detected using our device (Figure 7h). Although the signal morphology is not a perfect pulse wave curve due to the small variation of the applied force on the sensor, the function of counting pulses will not be affected due to the signal conditioning circuits.

## 4. Conclusions

In summary, an ultra-sensitive flexible capacitive pressure sensor with a pyramidal microstructured electrode is investigated. In comparison of sensors with the same dimensional microstructures on the dielectric layer, the sensitivity of the proposed device increased significantly from 3.1 to 70.6 kPa^−1^, and a detection limit of 1 Pa was achieved. FEA for hyperelastic materials reveals that the microstructures on electrode is critical to achieve high sensitivity. The influence of the duty ratio of the micro-pyramids on the sensitivity of the sensor is analyzed by both simulation and experiments. The durability and robustness of the device were also demonstrated by pressure testing for 10,200 cycles. Although gold is used as the electrode material in this work, any conductive material that can form thin film on the microstructures uniformly can be used in the proposed device. The proposed flexible pressure sensor with high sensitivity in the lower zone of the subtle-pressure regime exhibits enormous potential in human–electronics interfaces, electronic skin, and bio-monitoring devices.

## Supplementary Materials

The following are available online at https://www.mdpi.com/1424-8220/20/2/371/s1, Figure S1: The measurement of stress-strain curve for 10:1 PDMS, Figure S2: (a) Single pyramid microstructure capacitive sensor, (b) boundary conditions of the model, (c) the nonlinear mesh grid at the tip of the pyramid. Where *b_p_* is the width of the micro-pyramid, *l_p_* is the side length of the square electrode plate, *h_p_* is the electrode distance.
