# Supplementary material for: Ultra-Sensitive Flexible Pressure Sensor Based on Microstructured Electrode
Source: Sensors (Basel). 2020 Jan 9;20(2):371. doi: 10.3390/s20020371 (PMC7013555; doi:10.3390/s20020371)
Supplement: Supplementary file 1 [file sensors-20-00371-s001.pdf]

Supplementary Materials

Ultra-sensitive flexible pressure sensor based on microstructured electrode

Mengmeng Li [1,‡], Jiaming Liang [2,‡], Xudong Wang [1] and Min Zhang [1,*]

1 Shenzhen International Graduate School, Tsinghua University, Shenzhen, 518055, China; li-mm17@mails.tsinghua.edu.cn (M.L.); wangxd15@mails.tsinghua.edu.cn (X.W.)

2 Tsinghua-Berkeley Shenzhen Institute, Tsinghua University, Shenzhen, 518055, China; liangjm16@mails.tsinghua.edu.cn

* Correspondence: zhang.min@sz.tsinghua.edu.cn

‡ These authors contributed equally to this work.

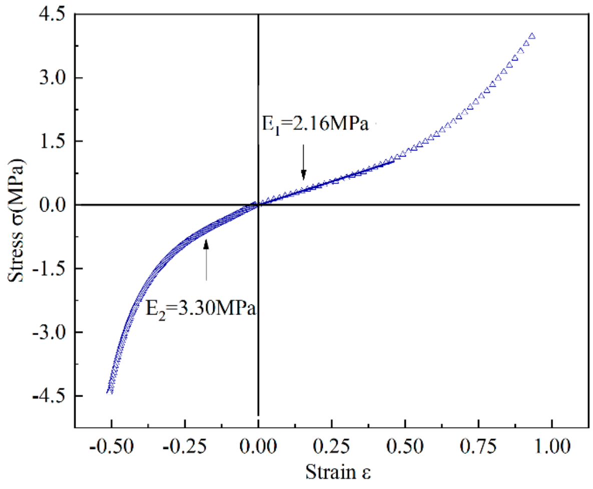

Figure S1 The measurement of stress-strain curve for 10:1 PDMS

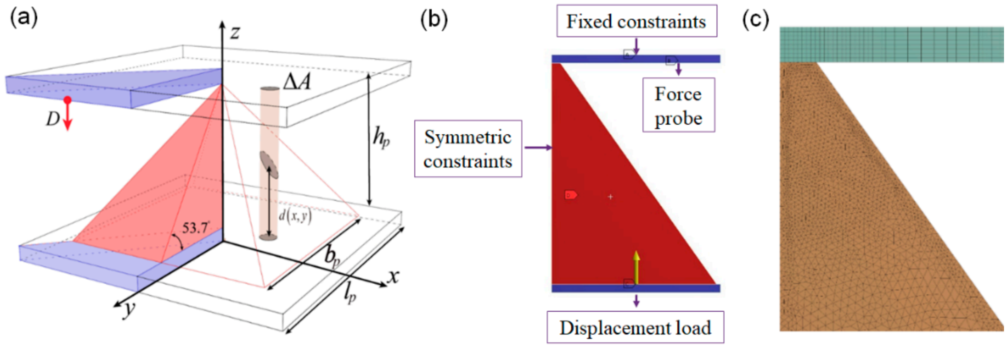

Figure S2 (a) Single pyramid microstructure capacitive sensor, (b) boundary conditions of the model, (c) the nonlinear mesh grid at the tip of the pyramid. Where b_p is the width of the micro-pyramid, l_p is the side length of the square electrode plate, h_p is the electrode distance.
